# Self-paced part-list cuing

**DOI:** 10.3758/s13423-021-01910-3

**Published:** 2021-07-09

**Authors:** Lisa Wallner, Karl-Heinz T. Bäuml

**Affiliations:** Department of Experimental Psychology, Regensburg University, 93040 Regensburg, Germany

**Keywords:** Memory, Episodic memory, Recall, Retrieval cues, Part-list cuing

## Abstract

Ironically, the presentation of a subset of studied material as retrieval cues at test often impairs recall of the remaining (target) material—an effect known as part-list cuing impairment. Part-list cues are typically provided at the beginning of the recall period, a time when nearly all individuals would be able to recall at least some studied items on their own. Across two experiments, we examined the effects of part-list cuing when student participants could decide on their own when the cues were presented during the recall period. Results showed that participants activated the cues relatively late in the recall period, when recall was already close to asymptote. Critically, such delayed cuing no longer impaired recall performance. The detrimental effect of part-list cuing, as it has been demonstrated numerous times in the memory literature, thus seems to depend on presentating the cue items (too) early in the recall period.

## Introduction

Bothlaboratory and applied memory research has demonstrated that episodic remembering can benefit enormously from the presence of adequate retrieval cues. Recall of a categorized list can be improved if the items’ category names are provided as retrieval cues at test (Tulving & Pearlstone, 1966); recall can benefit when an individual’s environment or mood during test matches the individual’s environment and mood during study (Godden & Baddeley, 1975; Teasdale & Fogarty, 1979); and recall of autobiographical events can benefit if the individual is told what the event was or where and when it happened (Wagenaar, 1986). All these findings converge on the view that retrieval cues may generally be beneficial, or at least neutral, for recall performance.

Research on so-called part-list cuing contrasts with this view, however. Over the past five decades, it has repeatedly been shown that when people study a list of items and, shortly after study, are provided with some of the items on that list as retrieval cues at test, they often do more poorly at recalling the remaining items on the list than do people who recall the items in the absence of such retrieval cues (Roediger, 1973; Slamecka, 1968). Although initially dismissed as a procedural artifact, such part-list cuing impairment has proven to be a very robust effect and to emerge in a variety of experimental settings (for reviews, see (Nickerson, 1984), or (Lehmer & Bäuml, 2018a)). The finding challenges the view that retrieval cues are generally beneficial, or at least neutral, for recall performance.

Numerous explanation for part-list cuing impairment have been suggested in the literature, but three accounts emerged as particularly promising to explain the effect. The first two accounts—blocking and inhibition—assume that part-list cuing induces covert retrieval of the cue items during recall, which, like selective retrieval practice can do in retrieval-induced forgetting (see (Anderson et al., 1994)), then blocks or actively inhibits recall of the remaining items (Bäuml & Aslan, 2004; Roediger, 1973). The third account—strategy disruption—claims that individuals create serial retrieval plans during study and the presentation of part-list cues at test then disrupts these strategies, which can impair recall performance (Basden & Basden, 1995). The three mechanisms are nonexclusive and different mechanisms may mediate part-list cuing impairment in different encoding situations (e.g., (Aslan & Bäuml, 2007; Bäuml & Aslan, 2006)).

In nearly all studies on the detrimental effects of part-list cuing, the test occurred shortly after study and part-list cues were provided at the beginning of the recall period. Likely, at this time of the recall period, most people would not have been in need of retrieval cues, because, given the short delay between study and the beginning of the test, they would have been able to recall at least some of the studied items on their own. People might regard cues more helpful later in the recall period, when recall is getting harder and may approach recall asymptote. At this point, people may expect part-list cues to provide access to further, not-easily-recalled items and thus improve recall performance. To date, no study has examined the effects of part-list cuing, if participants can decide on their own when the cues are provided during the recall period. This study examined such self-paced part-list cuing, expecting that, on average, individuals would not activate the cues at a very early stage of the recall period.

The time when participants activate part-list cues at test may indeed influence the effects on recall performance. For instance, if the covert retrieval of the cue items mediated the typical detrimental effect of part-list cuing, then blocking and inhibition should impair recall when the cues are provided at the beginning of the recall period, but this effect should be reduced or be even eliminated if the cues were activated later in the recall period. In fact, such late cuing would shield the already recalled items from the effects of blocking and inhibition and induce such effects for the not- yet-recalled items only, thus attenuating the overall detrimental effect of part-list cuing. Similarly, in situations in which strategy disruption mediates the detrimental effect, cuing later in the recall period would allow people to employ their own retrieval strategy at the beginning of the recall period, and would disrupt their strategy only later in the period, again reducing the overall detrimental effect. Thus, regardless of which detrimental mechanism operated in a particular situation, activating part-list cues later in the recall period should attenuate, or even eliminate, the detrimental effect on recall performance.

The results of two experiments are reported in each of which participants studied a list of unrelated items and were later asked to recall half of the items of the list, referred to as the target items. There were two part-list cuing conditions and a no-part-list cuing control condition in each experiment. In the standard part-list cuing condition, participants recalled the target items in the presence of the other half of the list items, which were provided at the beginning of the recall period to serve as retrieval cues for the target items. In the self-paced part-list cuing condition, participants could activate the same cue items on their own and do so whenever they liked during the recall period. Finally, in the control condition, participants recalled the target items in the absence of the cue items. Experiments 1 and 2 differed in whether the participants in the self-paced condition could activate the part-list cues as one single package (Experiment 1) or as three separate smaller packages distributed over the recall period (Experiment 2). The results of the two experiments will indicate whether self-paced part-list cuing, relative to standard part-list cuing, influences the time when the cue items are provided and thus also influences recall levels. Naturally, the experiments are not intended to explore the relative merits of the three accounts—blocking, inhibition, and strategy disruption—that are typically proposed to explain part-list cuing impairment.

## Experiment 1

### Method

#### Ethical considerations

All reported studies were carried out in accordance with the provisions of the World Medical Association Declaration of Helsinki.

#### Participants

A total of 144 students of Regensburg University participated in the experiment (*M*= 22.56 years, range = 18–34 years, 68.1% female, 31.3% male, and 0.7% diverse). They were equally distributed across the three between-participants conditions, resulting in *n* = 48 participants in each condition. We determined the desired sample size based on reported effect sizes in prior part-list cuing work from our lab (Bäuml & Schlichting, 2014; Lehmer & Bäuml, 2018b), counterbalancing purposes, and the results of an analysis of test power conducted with the G*Power program (version 3, (Faul et al., 2007)). For this analysis, we set alpha at .05, power at .95, and d at 0.70. All participants spoke German as native language and received monetary reward or course credit for participation.

#### Materials

Two lists of items were employed as study material, each consisting of 24 unrelated concrete German nouns. The lists were compiled from prior part-list cuing studies (Aslan & Bäuml, 2007; Lehmer & Bäuml, 2018b). Within each list, no two items had the same initial letter. For each of the two lists, two sets of target and cue items were constructed. For each set, 12 items of the list were randomly selected to serve as target items, with the remaining 12 items serving as the cue items. The distinction between target and cue items was unknown to the participants.

#### Design

The experiment had a unifactorial design with the between-participants factor of cuing (standard part-list cuing, self-paced part-list cuing, no part-list cuing). Participants in the standard part-list cuing condition were provided the 12 cue items at the beginning of the recall period, whereas participants in the self-paced part-list cuing condition could activate the same 12 cue items anytime during the recall period. In the no-part-list cuing condition, participants were not provided with any part-list cues. Assignments to part-list cuing conditions, lists, and list sets were counterbalanced across participants.

#### Procedure

In the study phase, the items of a list were exposed on a computer screen for 3.5 s per item and in a random order. The study phase was followed by a 2-min distractor phase, in which participants solved arithmetic problems as a recency control. At test, all participants received a sheet of paper with the unique initial letters of the 12 target items, presented in two columns of six items each and in a random order. Participants were asked to fill in the missing target items in any order they wished for the next 2 min. Prior to this 2-min recall period, participants in the standard part-list cuing condition were provided the 12 cue items in three columns of four items each on the computer screen. The order of the items was random. Participants were told to use these items as retrieval cues for recall of the list’s target items. The cue items were presented sequentially at a 2 s rate and participants were asked to read each item aloud. Presented items remained on the screen until the end of the recall period. The self-paced part-list cuing condition was identical to the standard part-list cuing condition, with the only difference that participants could determine when during the recall period (and if) the 12 cue items were provided. Participants were told that they could push a button on the computer keyboard, which initiated presentation of the list’s remaining 12 items on the computer screen; these items could serve as retrieval cues for recall of the target items and would stay present on the screen for the rest of the recall period. Cue presentation time (12*2 s) was discounted from duration of the recall period. In the no-part-list cuing condition, no cue items were presented.

### Results

Forty-six of the 48 participants in the self-paced part-list cuing condition activated the part-list cues. Mean activation time for the 46 participants was 49.94 s (95*%* *CI* = [42.02,57.86]). A rough breakdown of activation times shows that 15 participants activated the cues within the first 30 s, 32 participants within the first 60 s, and 40 participants within the first 90 s (see Table 1).

**Table 1:** Numbers of participants in the self-paced part-list cuing condition activating the 12-item cue package (Experiment 1) and each of the three 4-item cue packages (Experiment 2) in single intervals of the recall period

Intervals of the recall period	1–30 s	31–60 s	61–90 s	91–120 s
Experiment 1				
(Single) Cue package	15	17	8	6
Experiment 2				
Cue package 1	14	20	10	3
Cue package 2	0	11	15	15
Cue package 3	0	3	9	13

**Fig. 1:**
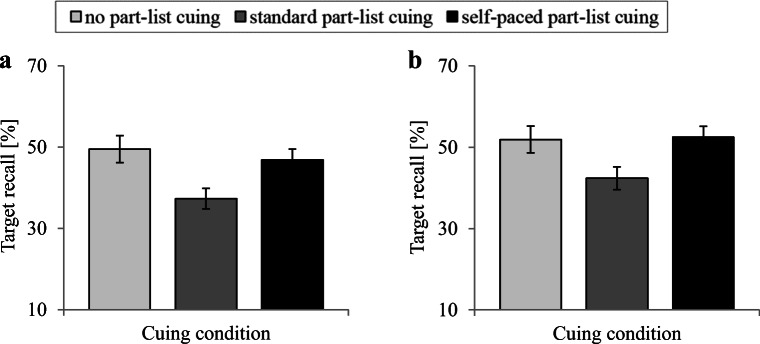
Results of Experiment 1**(a)** and Experiment 2**(b)**. Percentage of correctly recalled target items is shown as a function of cuing condition (no part-list cuing, standard part-list cuing, self-paced part-list cuing). *Error bars* represent ± 1 standard error

Figure 1a depicts mean recall levels for the target items as a function of cuing condition. An unifactorial analysis of variance (ANOVA) with the between-participants factor of cuing (no part-list cuing, standard part-list cuing, self-paced part-list cuing) showed a significant effect of condition, *F*(2,141) = 5.08, *MSE* = 387.15, *p* = .007, *η*^2^ = 0.07. Follow-up comparisons revealed that standard part-list cuing impaired target recall relative to the no-part-list cuing condition (standard: *M* = 37.33*%*, 95*%* *CI* = [32.24,42.42]; control: *M* = 49.48*%*, 95*%* *CI* = [42.79,56.17]), *t*(94) = 2.91, *p* = .005, *d* = 0.59, whereas target recall in the self-paced part-list cuing condition did not differ from recall in the no-part-list cuing condition (self-paced: *M* = 46.87*%*, 95*%* *CI* = [41.65,52.10]), *t*(94) < 1, *d* = 0.13. Consistently, target recall in the self-paced condition was significantly higher than in the standard condition, *t*(94) = 2.63, *p* = .010, *d* = 0.54.

### Discussion

As expected, most participants in the self-paced part-list cuing condition did not activate the part-list cues particularly early in the recall period, which contrasts with the standard part-list cuing condition, in which the cue items were provided at the beginning of the recall period. This difference in the timing of part-list cuing was accompanied by a marked difference in recall performance between the two cuing conditions, with higher recall in the self-paced than the standard part-list cuing condition. Moreover, recall in the self-paced condition was similar to recall in the no-part-list cuing condition, indicating that the typical detrimental effect is eliminated when cuing is self-paced. Together, the findings on cue activation times and recall levels suggest that participants in the self-paced condition activate the cue items sufficiently late in the recall period to shield (most of) the target items from the potentially detrimental effects of blocking, inhibition, or strategy disruption. The goal of Experiment 2 was to replicate the results of Experiment 1 with other material and a self-paced cuing condition, in which participants could activate several, but smaller, cue packages.

## Experiment 2

### Method

#### Participants

Another 144 students of Regensburg University took part in the experiment (*M*= 22.17 years, range, 18-38 years, 72.9% female, 27.1% male). Sample size followed Experiment 1. Again the participants were equally distributed across the three between-participants conditions, resulting in *n* = 48 participants in each condition. All participants spoke German as native language and received monetary reward or course credit for participation.

#### Materials

Two new lists were employed in this experiment, again consisting of 24 unrelated concrete German nouns. They were compiled from prior part-list cuing studies ((Aslan & Bäuml, 2007); (Lehmer & Bäuml, 2018b)). Within each list, no two items had the same initial letter. For each of the two lists, again two sets of 12 randomly selected target and 12 remaining cue items were constructed. Like in Experiment 1, classification of items into target and cue items was unknown to the participants.

#### Design and procedure

Design and procedure were identical to Experiment 1, with the only difference that the procedure in the self-paced part-list cuing condition differed from the procedure in Experiment 1. Participants could activate three cue packages of four items each whenever they liked during the recall period. Participants were told that they had the chance to receive three packages of four list items each as retrieval cues and that they could determine the time of presentation of each cue package on the computer screen by pushing a button on the computer keyboard, which initiated presentation of the package; the cue items would stay present on the screen for the rest of the recall period. If participants pushed the activation button of the computer keyboard for the first time, the first four–randomly selected—cue items were activated. They were presented successively at a 2-s rate per item in a left column of the computer screen. Participants were asked to read each item aloud. If participants pushed the activation button a second and third time, two further packages of four cue items were activated and the corresponding items were presented in a random order in a middle column (second package) and a right column (third package) of the computer screen. For each cue package, cue presentation time (4*2 s) was discounted from duration of the recall period. All presented cue items remained on the screen until the end of the recall period.

### Results

Forty-seven of the 48 participants in the self-paced part-list cuing condition activated at least one cue package, 41 participants activated at least two cue packages, and 25 participants activated all three cue packages. Mean activation time was 49.11 s (95*%* *CI* = [41.49,56.72]) for the first cue package, 78.24 s (95*%* *CI* = [71.04,85.45]) for the second cue package, and 88.56 s (95*%* *CI* = [79.36,97.76]) for the third cue package. A rough breakdown of activation times shows that 14 participants activated the first cue package within the first 30 s, 34 participants within the first 60 s, and 44 participants within the first 90 s (see again Table 1), which is highly similar to cue activation times for the single cue package in Experiment 1.

Figure 1b shows mean recall levels for the target items as a function of cuing condition. An unifactorial ANOVA with the between-participants factor of cuing (no part-list cuing, standard part-list cuing, self-paced part-list cuing) showed a significant effect, *F*(2,141) = 3.77, *MSE* = 411.02, *p* = .026, *η*^2^ = 0.05. Follow-up comparisons revealed that standard part-list cuing impaired target recall compared to the no-part-list cuing condition (standard: *M* = 42.36*%*, 95*%* *CI* = [36.74,47.98]; control: *M* = 51.90*%*, 95*%* *CI* = [45.27,58.53]), *t*(94) = 2.21, *p* = .030, *d* = 0.45, whereas self-paced part-list cuing left recall largely unaffected relative to the no-part-list cuing condition (self-paced: *M* = 52.47*%*, 95*%* *CI* = [47.13,57.80]), *t*(94) < 1, *d* = 0.03. Again, target recall in the self-paced condition was significantly higher than in the standard condition, *t*(94) = 2.62, *p* = .010, *d* = 0.54.

### Discussion

Like in Experiment 1, most participants in the self-paced part-list cuing condition did not activate part-list cues very early in the recall period. Nearly all participants activated at least one cue package, more than 80% of the participants activated at least two cue packages, but only about 50% of the participants made use of the third cue package. Part-list cuing in the self-paced condition thus differed considerably from that in the standard condition. This difference was again reflected by a marked difference in recall performance between the two conditions, with higher recall in the self-paced than the standard cuing condition. Recall in the self-paced condition was even similar to recall in the no-part-list cuing condition, suggesting that the typical detrimental effect was eliminated by self-paced cuing. Together with the findings on cue activation times, results are again consistent with the view that participants in the self-paced condition activated cue items sufficiently late in the recall period to protect target items from the potentially detrimental effects of part-list cuing.

This view on self-paced part-list cuing suggests that recall in Experiments 1 and 2 was already fairly close to asymptote when participants activated cues. In a final study, we tested this proposal more directly by measuring participants’ cumulative recall for the experimental setup employed in the no-part-list cuing condition of Experiment 1. Cumulative recall was measured after 30, 60, 90, and 120 s of the recall period and an exponential function was fit to the recall data. The function will indicate how close participants’ recall was to asymptote when they activated the cue items in Experiment 1.

## Study on cumulative recall

### Method

#### Participants

Another 48 students of Regensburg University took part in the experiment (*M*= 22.23 years, range, 18-28 years, 83.3% female, 16.7% male). Sample size followed Experiment 1. All participants spoke German as native language and received monetary reward or course credit for participation.

#### Materials

Materials were identical to Experiment 1.

#### Procedure

The procedure was largely identical to the no-part-list cuing condition of Experiment 1, with the only difference that participants’ recall was measured after 30, 60, 90, and 120 s of the recall period. At the beginning of the test, participants received a sheet of paper with initial letters of the 12 target items presented in two columns of six items each in a random order. Like in Experiment 1, participants were asked to fill in the missing target items in any order they wished for the next 2 min. Unlike in Experiment 1, participants were cautioned that during testing they would repeatedly be asked to change pencils but that they should try not to be diverted. Participants started target recall with a blue pencil. After 30 s, they were given a green pencil, after 60 s a red pencil, and after 90 s a black pencil to continue with target recall. This procedure allowed measurement of participants’ recall levels after each of the four selected time intervals.

The cumulative recall data as measured 30, 60, 90, and 120 s after the beginning of the recall period were fit by an exponential function, *F*(*t*) = *N*(1 − *e*^−*t*/*τ*^), where *F*(*t*) represents the cumulative number of items recalled by time *t*, *N* represents asympotic recall, i.e., the estimated percentage of items that could be produced given unlimited time, and *τ* represents mean response latency to recall those N items (e.g., (Bousfield & Sedgewick, 1944; Roediger, 1978; Rohrer & Wixted, 1994)). The best fitting exponential was determined by least squares minimization.

### Results

**Fig. 2:**
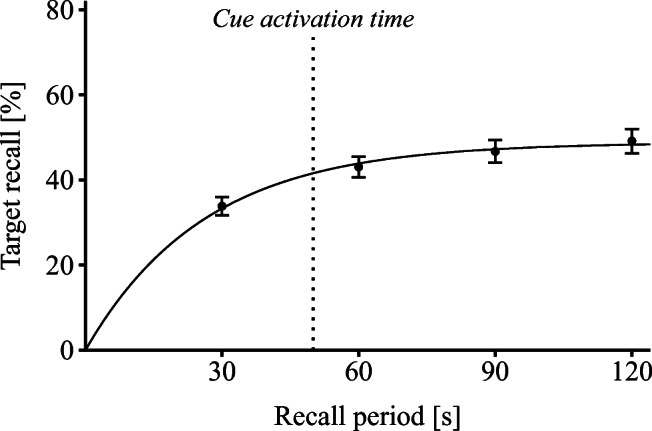
Results of the study on cumulative recall. Percentage of correctly recalled target items after 30, 60, 90, and 120 s is shown together with the best-fitting exponential function. Cue activation time is taken from Experiment 1. *Error bars* represent ± 1 standard error

Cumulative target recall was 33.85% (95*%* *CI* = [29.55, 38.16]) after 30 s, 43.06% (95*%* *CI* = [38.16,47.95]) after 60 s, 46.70% (95*%* *CI* = [41.35,52.05]) after 90 s, and 49.13% (95*%* *CI* = [43.35,54.92]) after 120 s. The observed recall level after 120 s was nearly identical to the recall level observed in the no-part-list cuing condition of Experiment 1 (*M* = 49.48*%*, 95*%* *CI* = [42.79,56.17]), *t*(94) < 1, *d* = 0.02. Figure 2 shows the four recall levels together with the best-fitting exponential. The exponential described the data well and accounted for 99% of the variance in the data. Parameter estimates were 25.86 for parameter *τ* and 48.71 for parameter *N*, which is close to the recall level observed after 120 s and indicates that participants would not have been able to recall many more items if the recall period had been prolonged.

Experiment 1 found a mean cue activation time of 49.94 s. On the basis of the exponential fit, we calculated a cumulative recall level of 41.56% for this cue activation time. Although this value is slightly below the estimated asymptotic recall level, it shows that 85% of the recallable items were already recalled at this point in time. This value is sufficiently close to asymptote to support the view that participants activated the cue items in Experiments 1 and 2 late enough to shield most (recallable) target items against the detrimental influences of part-list cuing.

## General discussion

This study is the first to investigate the effects of self-paced part-list cuing on recall performance. Allowing participants to activate part-list cues whenever they liked during the recall period, we found that most participants did not activate the cues early in the recall period. Rather, it took about 50 s on average until the participants activated cues items. This finding arose both when the cues could be activated as a single package (Experiment 1) and when the cues could be activated in a more sliced manner (Experiment 2). Self-paced part-list cuing thus created a cuing situation that sharply contrasts with the situation in typical part-list cuing studies, in which (all) part-list cues are presented right at the beginning of the recall period.

Self-paced part-list cuing did not only influence the time when the cue items were provided, it also influenced recall levels. Whereas we found the typical detrimental effect of part-list cuing in the standard cuing condition, we found recall levels in the self-paced cuing condition to be similar to recall levels in the absence of part-list cues, indicating that self-paced cuing is beneficial for recall performance relative to the standard cuing condition. The detrimental effect of part-list cuing in the standard cuing condition has been attributed to covert retrieval of the cue items—and induced blocking or inhibition processes—or to strategy disruption. All these processes, however, should influence recall only if the cues were provided fairly early in the recall period before a substantial number of target items has already been recalled, which is the procedure with standard part-list cuing. With self-paced part-list cuing, the cuing occurs much later in the recall period, when recall is already close to asymptote. At this time, many of the target items have already been recalled and thus are protected against the detrimental effects of part-list cuing.

Slamecka (1968) started part-list cuing research with the idea in mind that part-list cuing may actually improve recall performance. Such improvement should arise because associations between items were assumed to be built up during study and the cue items should then serve as effective retrieval cues for associated items. Part-list cuing research over the past five decades has challenged this idea by typically reporting detrimental rather than beneficial effects of part-list cuing. The present results challenge the idea even further by showing that potential beneficial processes of part-list cuing do not even show up when recall is already close to asymptote and the cue items might help accessing target items not recalled in the absence of the cue items.^1^ Thus, with the typical setup used in most part-list cuing studies, there is no evidence for a contribution of beneficial processes to recall performance.

The present experiments tested student participants and used material and procedure as they have typically been employed in prior research on part-list cuing. With this setting, self-paced part-list cuing can obviously circumvent the detrimental effect that is typically found when part-list cues are provided by the experimenter at the beginning of the recall period. However, future work should address a few generalizability issues. Two such issues stand out. The one issue is whether results will extend to more complex study material and arise also outside the lab, like in educational or eyewitness testimony situations, when cues are used to improve a student’s or a witness’s recall of some target material. The other issue is whether results will generalize to individuals younger or older than the present student participants. Indeed, both children and older adults often show impaired memory function and thus may tend to activate cues earlier than the young adults did in the present study. Knowing whether age influences the beneficial effect of self-paced cuing therefore is a high priority for future work in this research area.

## Conclusions

Retrieval cues are generally regarded as beneficial, or at least neutral, for recall performance—a view of considerable relevance for both memory theory and application. Findings from part-list cuing research over the past five decades have challenged this view, however, demonstrating that, if a subset of studied material is provided as retrieval cues at the beginning of a recall test, recall of the remaining (target) material is often impaired. This study shows that this core finding does not generalize to self-paced part-list cuing, when individuals can decide on their own when during the recall period the cues are provided. The potentially detrimental effects of cues on recall may thus be circumvented with self-paced cuing.

## Footnotes

1 This finding is consistent with an observation by Slamecka (1968). In his Experiment V, participants engaged in free recall of a previously studied word list for 4 min, had their recall checked for 1 min, and then were presented half of the unrecalled words as cues for the other unrecalled words. Slamecka found that the part-list cues given after 4 min of recall did not boost recall relative to a control condition that used the same procedure, except that no cues were provided.
